# Neuromyelitis optica spectrum disorder in an 11-year-old: a case report in a pediatric patient from Nepal

**DOI:** 10.1097/MS9.0000000000004760

**Published:** 2026-01-20

**Authors:** Prakash Thapa, Sriram Kc, Bulbul Pradhan, Inesh Khanal, Aakripa R. Shrestha

**Affiliations:** a Department of Pediatrics, Patan Academy of Health Sciences, Lagankhel, Lalitpur, Nepal; b Department of Pediatrics, Nepal Armed Police Force Hospital, Kathmandu, Nepal; c Department of Pediatrics, Patan Academy of Health Sciences, Lagankhel, Lalitpur, Nepal; d School of Medicine, Patan Academy of Health Sciences, Lagankhel, Lalitpur, Nepal

**Keywords:** AQP4, case report, Devic’s syndrome, neuromyelitis optica spectrum disorder, pediatric neurology

## Abstract

**Introduction and importance:**

Neuromyelitis optica spectrum disorder (NMOSD) is an auto-immune inflammatory disorder of the central nervous system mediated by auto-antibodies against Aquaporin-4 (AQP4). It causes vision impairment, vomiting, and paralysis.

**Case presentation:**

We report a case of an 11-year-old Nepalese girl who initially presented with persistent vomiting for 2 months, followed by progressive limb weakness and facial asymmetry. She was initially misdiagnosed as gastroduodenitis with mesenteric lymphadenitis and later with major depressive illness and dissociative motor disorder. Neurological examination revealed multiple cranial nerve palsies and upper motor neuron signs. Magnetic resonance imaging (MRI) of the brain and cervical spine demonstrated long segment T2 hyper-intensities involving the medulla, cervicomedullary junction, and cervical spinal cord which was highly suggestive of transverse myelitis with postrema lesions. Serum anti-AQP4 antibody testing done by indirect immunofluorescence assay was positive which confirmed NMOSD. The patient was treated with intravenous methylprednisolone pulse therapy followed by intravenous immunoglobulin and transitioned to oral prednisolone with gradual improvement in neurological function.

**Clinical discussion:**

This case highlights diagnostic challenge of NMOSD, particularly when presented with area postrema syndrome without early neurological findings. Such atypical presentations are frequently mistaken for gastrointestinal or psychiatric conditions, delaying appropriate diagnosis and management. MRI findings and a positive anti-AQP-4 antibody remain key to confirming the diagnosis. Early initiation of immunotherapy is essential to prevent irreversible neurological deficits.

**Conclusion:**

NMOSD should be considered in children presenting with unexplained persistent vomiting and subsequent neurological deficits. A high index of suspicion, prompt antibody testing, and timely immunotherapy are vital for favorable outcomes.

## Introduction

Neuromyelitis optica spectrum disorder (NMOSD) is an auto-immune inflammatory disorder of the central nervous system (CNS) characterized by demyelination and axonal damage of spinal cord and optic nerve, causing vision impairment, vomiting and paralysis^[1,2]^. It is due to auto-antibodies against Aquaporin-4 (AQP4), a protein abundant in the astrocytic foot processes of blood-brain barrier and in spinal cord gray matter, periaqueductal, and periventricular regions^[3–5]^.

The incidence and prevalence for NMOSD in children ranged from 0.01–0.06/100 000 and 0.06–0.22/100 000 respectively, with a female predominance[6]. Here, we report a case of an 11-year-old Nepalese girl diagnosed with NMOSD. We report the case by remaining complaint with the Transparency In The reporting of Artificial Intelligence (TITAN) Guidelines 2025, governing declaration and use of Artificial Intelligence[7].

## Case report

### Initial presentation

An 11-year-old female from Surkhet presented with vomiting for 2 months, upper and lower limb weakness for 12 days, and facial asymmetry for 2 days. She initially developed multiple episodes of vomiting and was admitted to a hospital in Surkhet and treated with a provisional diagnosis of acute gastritis. Vomiting subsided, and she was discharged on proton pump inhibitors. After 10 days, she was readmitted to the same center due to persistent vomiting unresponsive to antiemetics. Ultrasonography of abdomen and pelvis revealed mesenteric lymphadenitis, and upper gastroduodenoscopy revealed duodenitis. She was discharged after 14 days when symptoms subsided.

After 10 days of discharge, she was admitted to a tertiary center in Kathmandu for persistent vomiting, lethargy, and speech difficulties for 3 days. Organic causes were ruled out, psychiatric evaluation was done, and she was diagnosed with major depressive illness (MDI). She was referred to a psychiatric hospital. After 3 days of admission, she developed left lower limb weakness which gradually progressed to bilateral upper and lower limbs, with equal proximal and distal involvement with intact sensation. A diagnosis of Dissociative Motor Disorder (DMD) was made, and Quetiapine and Sertraline were started. After 5 days of treatment, she developed left-sided facial deviation. There was difficulty in speech and closing the right eye. She was then referred to Patan Hospital for further management.

### Hospital course

After 10 days of discharge, she was again admitted to a tertiary center in Kathmandu for persistent vomiting, lethargy, and speech difficulties for 3 days. Organic causes were ruled out, psychiatric evaluation was done, and she was diagnosed with MDI. She was referred to a psychiatric hospital. After 3 days of admission, she developed left lower limb weakness which gradually progressed to bilateral upper and lower limbs, with equal proximal and distal involvement with intact sensation. A diagnosis of DMD was made, and Quetiapine and Sertraline were started. After 5 days of treatment, she developed left-sided facial deviation. There was difficulty in speech and closing the right eye. She was then referred to our center for further management.

### Neurological examination

The neurological status of the patient is as summarized in Table 1. The full timeline of the patient from the initial appearance of her symptoms to the treatment and outcome is highlighted in Table 3.

**Table 1:** Neurological status of the patient

Domain	Findings	Interpretation/remarks
Mental status	Oriented to time, place, and person	Normal higher mental function
Attention, concentration, and memory	Intact	Normal cognitive function
Perception and thought	No delusions or hallucinations	Normal
Cranial nerves	III (Oculomotor): Right medial rectus palsy VII (Facial): Right-sided Bell’s palsy X (Vagus): Uvula deviated to left side XII (Hypoglossal): Tongue deviated to right side	Right III, VII, X, and XII nerve palsies
Motor system	Bulk: Normal in all limbs Tone: Increased in all muscle groups Power: 2/5 on right upper and lower limbs; 1/5 on left upper and lower limbs	Spastic weakness, more severe on the left side
Deep tendon reflexes	Biceps, triceps, supinator, knee, and ankle reflexes exaggerated bilaterally	Hyperreflexia suggestive of upper motor neuron lesion
Plantar reflex	Bilateral upgoing (extensor)	Upper motor neuron sign
Clonus	Bilateral ankle clonus present	Upper motor neuron sign
**Overall Impression**	Multiple cranial nerve palsies (III, VII, X, and XII) with bilateral pyramidal tract signs	Suggestive of brainstem lesion with bilateral upper motor neuron involvement

**Table 2:** Core clinical characteristics for diagnosis of NMOSD^8^

1	Optic neuritis
2	Acute myelitis
3	Area postrema syndrome: episode of otherwise unexplained hiccups or nausea or vomiting,
4	Acute brainstem syndrome
5	Symptomatic narcolepsy/acute diencephalic syndrome with NMOSD-typical diencephalic MRI lesions
6	Symptomatic cerebral syndrome with NMOSD-typical brain lesions

**Table 3:** Timeline diagram of the patient discussed in the case

Time	Event	Diagnosis/key finding
2 months before presentation	Persistent, multiple episodes of vomiting	Initial diagnosis of acute gastritis
10 days later	Persistent vomiting led to readmission to Surkhet Hospital	Duodenitis and mesenteric lymphadenitis found on imaging
Later hospital stay	Discharged after 14 days when symptoms subsided	Vomiting subsided after treatment
10 days after discharge	Readmitted to a tertiary center in Kathmandu with persistent vomiting, lethargy and speech difficulties for 3 days	Diagnosis with major depressive illness (MDI) after organic causes were ruled out
3 days after MDI diagnosis	Developed left lower limb weakness which gradually progressed to bilateral upper and lower limbs	Diagnosis with Dissociaive Motor Disorder (DMD)
5 days after DMD diagnosis	Developed left sided facial deviation, difficulty with speech and difficulty closing right eye	Differential diagnoses included NMOSD, ADEM, and MS
After transfer/neurological exam	Neurological exam revealed CN III, X and XII palsies and signs of upper motor neuron lesion (weakness 1/5-2/5, increased tone, brisk reflexes)	Differential diagnoses included NMOSD, ADEM, and MS
Imaging/workup	MRI showed long segment hyperintensity in the medulla, cervicomedullary junction, and cervical spine, suggestive of transverse inflammatory myelitis with postrema lesion	Started on pulse dose methylprednisolone (30 mg/kg/day for 5 days)
Confirmation	Anti- AQP4 antibodies tested positive by indirect immunofluorescence Assay	Definitive diagnosis of NMOSD
treatment	Started on intravenous immunoglobulin at 2 gm/kg over 5 days	Symptoms improved: left limb power improved to 3/5, speech improved, and vomiting subsided
Discharge	Discharged after 26 days	Discharged on oral prednisolone (1 mg/kg/dose) with a plan for gradual tapering over 6 months.

### Diagnosis

The differential diagnoses of demyelinating disorders like NMOSD, acute disseminated encephalomyelitis (ADEM), and multiple sclerosis (MS) along with space occupying lesions were made. Blood, CSF (Cerebrospinal Fluid), and neuroimaging were sent. Blood and CSF analysis was normal. Magnetic resonance imaging (MRI) T2-weighted short-tau inversion recovery (T2w-STIR) revealed long segment ill-defined non-enhancing hyperintensity in the medulla, the cervicomedullary junction, and the proximal aspect of the cervical spine suggestive of transverse inflammatory myelitis with postrema lesion

HIGHLIGHTS

**Figure 1.**
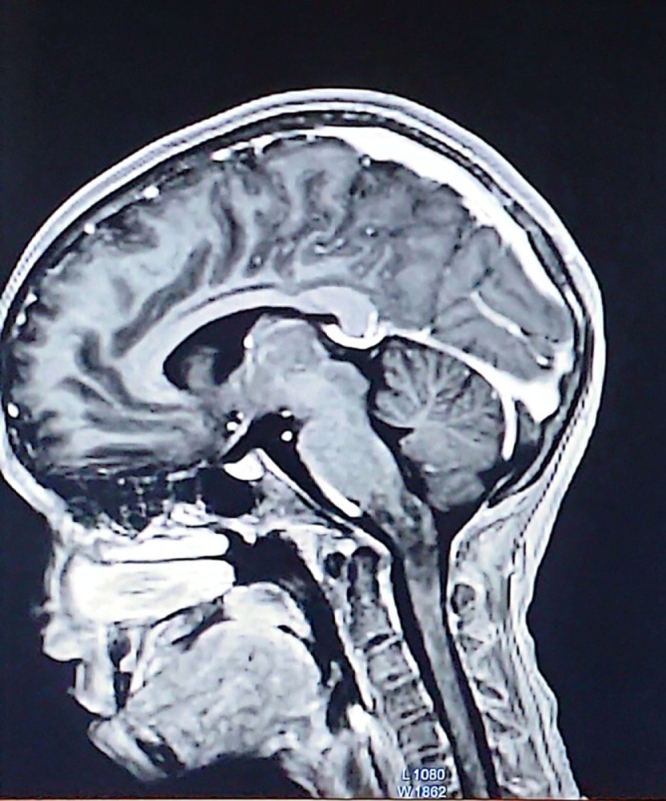
Non-enhancing hyperintensity in medulla and proximal aspect of cervical spine (T1-weighted image).

**Figure 2.**
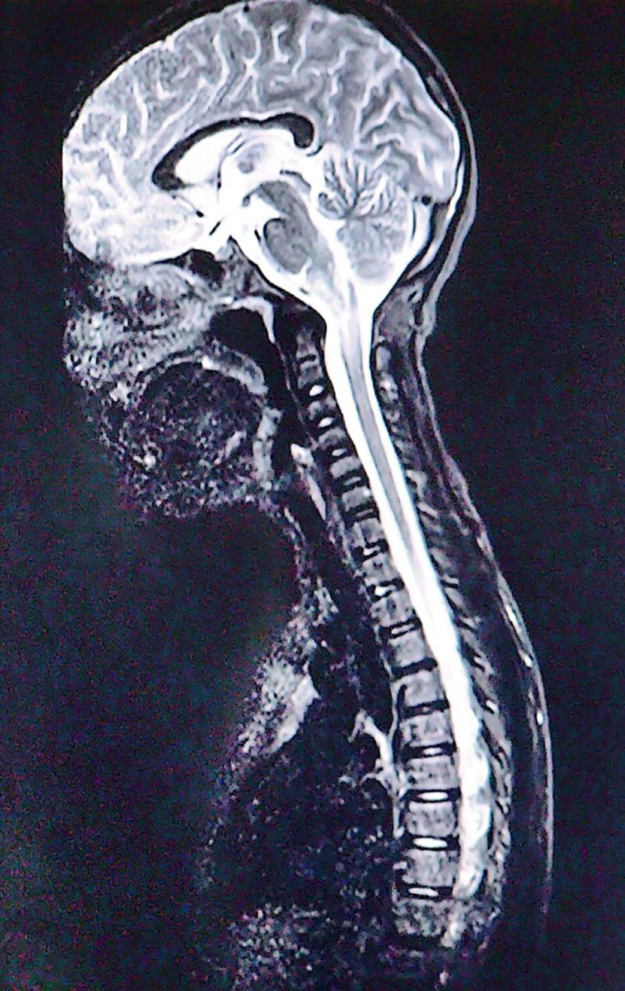
Postrema lesions extending into the medulla (T2w-STIR image).

**Figure 3.**
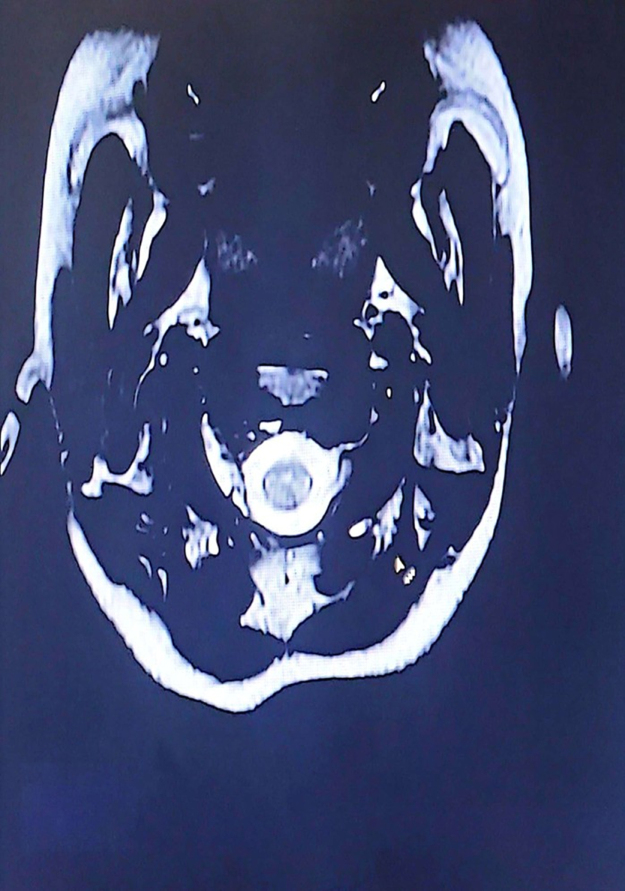
Transverse myelitis in the central cord at C1 vertebral level.

- Here are the key highlights from the case report of an 11-year-old Nepalese girl with neuromyelitis optica spectrum disorder (NMOSD):
- **Rare pediatric presentation in Nepal:** This is one of the first detailed reports of NMOSD in an 11-year-old from Nepal, expanding our understanding of geographic and age-specific manifestations of the disease.
- **Initial misdiagnoses:** The patient’s persistent vomiting led first to a diagnosis of gastroduodenitis, and then to psychiatric labels (major depressive illness and dissociative motor disorder) before neurological evaluation revealed NMOSD.
- **Core clinical features with delayed recognition:** Despite having area postrema syndrome (intractable vomiting) and evolving motor and cranial nerve deficits, the diagnosis was delayed – a reminder that isolated gastrointestinal or psychiatric symptoms may mask NMOSD.
- **Confirmatory investigations:** Magnetic resonance imaging showed longitudinally extensive transverse myelitis with area postrema involvement, and serum anti–Aquaporin-4 antibodies were positive, fulfilling international diagnostic criteria for NMOSD.
- **Prognostic and preventive considerations:** Long-term immunosuppression and close monitoring for relapse and treatment toxicity (e.g., steroid side effects) are essential to prevent further attacks and optimize outcomes.

(Fig. 1 and Fig. 2). The transverse myelitis was mainly involving the central cord (Fig. 3).

### Treatment and outcome

She was started on pulse therapy of injection methylprednisolone at 30 mg/kg/day for 5 days, while additional workup was being done. Anti-AQP4 antibodies tested by the indirect immunofluorescence assay were positive, so the diagnosis of NMOSD was made. Intravenous Immunoglobulin (IVIg) at 2 gm/kg was given over 5 days. After completing the course of immunoglobulins, oral prednisolone was started at 1 mg/kg/dose. With this treatment, the power of the left upper and lower limbs had improved to 3/5, and her speech improved and vomiting also subsided. She was discharged after 26 days, with oral dose of prednisolone and plan of gradual tapering over 6 months. She had marked improvement in her speech, and vomiting had subsided by the time of discharge. The patient was advised for close follow-up to monitor steroid toxicity.

## Discussion

NMOSD, originally known as Devic’s syndrome, is a disease of the CNS characterized by inflammatory attacks on the optic nerve and spinal cord, along with episodes of intractable vomiting and hiccups^[1,3]^. The hallmark of NMOSD is the presence of antibodies against AQP-4 which is predominantly expressed in CNS astrocytes, especially in the blood-brain barrier, ependymal cells, retina, and inner ear[3].

The diagnostic criteria of NMOSD with AQP-4, as per The International Panel for NMO Diagnosis, is the presence of at least one core clinical characteristic; positive test for anti-AQP4 antibodies and exclusion of alternative diagnosis clinically and radiologically[8]. The core clinical characteristics are mentioned in Table 2[8].

Optic neuritis is the most common presenting characteristic in pediatric AQP4-IgG seropositive NMOSD, manifesting in 43–47% of cases followed by myelitis in 24–36%, area postrema syndrome (APS) in 38%, symptomatic cerebral syndrome in 30%, and symptomatic narcolepsy/acute diencephalic syndrome in 3.8%[9].

In this case, despite the presence of core clinical symptoms at initial presentation, there was a delay in diagnosis. This could be because when APS occurs without neurological deficits, it is commonly misdiagnosed to be a disease of the gastrointestinal system[10]. There was a similar scenario in our case, where the patient had initially presented with persistent vomiting. The patient was initially diagnosed and treated on the line of gastroduodenitis. Then she developed neuro-psychiatric symptoms and was diagnosed with MDI and DMD. NMOSD can have psychiatric disturbances manifesting as moderate-to-severe depression and in pediatric patients functional neurological symptoms such as motor weakness or speech difficulty can sometimes be attributed to psychogenic disorders leading to diagnostic oversight[11]. In a retrospective review of medical records from six Latin American countries, frequency of misdiagnosis of NMOSD is relatively frequent (~12%), with MS being the most common incorrect diagnosis, often due to misinterpretation of clinical and neuroimaging findings[12].

After the gradual increase in the severity of neurological symptoms, neuroimaging was done. MRI features like cervical transverse myelitis extending into the medulla and area postrema lesion were highly suggestive of demyelinating disorders like NMOSD, ADEM, or MS^[8,13]^. The distinction hinges on the detection of anti-AQP4 antibodies that has a high specificity and sensitivity of 91 and 98%, respectively[14]. Anti-AQP4 was positive in this case. Anti-AQP4 antibody testing alone is not considered reliable for a definitive diagnosis of NMOSD because a significant number of patients with clinical features of NMOSD are seronegative for the AQP4 antibody[8]. This has led to the identification of a distinct subgroup of patients who test positive for other antibodies such as myelin oligodendrocyte glycoprotein (MOG-IgG)[15]. Additionally, the possibility of false-negative results due to immunosuppressant treatment or the use of less sensitive assays further highlights the need for comprehensive clinical assessment alongside serological testing[8]. Mimics of NMOSD should be excluded, and there should be presence of at least two core symptoms and highly suggestive MRI to label it as such[8].

The patient was started on pulse therapy of injection methylprednisolone at 30 mg/kg/day, while additional workup was being done. After diagnosis of NMOSD was made, IVIg at 2 gm/kg was given over 5 days. The patient was started and discharged on oral prednisolone at 1 mg/kg/dose with a plan of tapering over 6 months. This treatment plan is similar to a case report from London, for an acute attack of NMOSD[1]. IVIg helps in reducing ingoing auto-immune mediated damage to the CNS by blunting the anti-body and complement-driven inflammation[16]. In several neurological conditions such as NMOSD, GBS, CIDP, and Myasthenia Gravis, it helps in the modulation of abnormal immune responses[16]. If improvement is not seen then, five cycles of plasma exchange is recommended[1]. Long-term immunosuppressants with oral prednisolone is required to prevent further attacks.^1^ Newer therapies including human monoclonal antibodies like Eculizumab, Rituximab, Satralizumab, and Tocilizumab are being considered[3].

## Conclusions

NMOSD is a rare and life threatening but treatable demyelinating disease requiring a high index of suspicion for correct diagnosis and treatment. For early recognition and treatment, a multidisciplinary approach to treatment is needed due to the wide ranging symptoms affecting various organ systems.

## Learning points

- NMOSD can present atypically in children, often presenting with unexplained vomiting or hiccups which can initially be misdiagnosed as gastrointestinal disorders.
- Symptoms like speech difficulty or motor weakness may be mistakenly attributed to neuropsychiatric disorders. Due to overlapping neuropsychiatric features, it causes significant diagnostic delay.
- A high clinical suspicion is essential for diagnosis. The key features of NMOSD include: (MRI findings) long- segment transverse myelitis and area postrema lesions extending into the medulla, serological testing for anti-AQP4 antibodies.
- Acute attacks are treated with high dose intravenous methylprednisolone pulse therapy and often IVIg.

- Long-term immunosuppression (e.g., oral prednisolone with gradual tapering) is required to prevent future relapses.

## Data Availability

Not applicable.
